# Providers’ perspectives of socio-cultural and health service challenges related to postpartum family planning in Alexandria, Egypt

**DOI:** 10.1186/s42506-020-00066-7

**Published:** 2021-02-17

**Authors:** Heba Mahmoud Taha El Weshahi, Ahmed Fawzy Galal, Eman Anwar Sultan

**Affiliations:** 1 Community Medicine Department, Faculty of Medicine, Alexandria University, Alexandria, Egypt; 2 Obstetrics and Gynecology Department, Faculty of Medicine, Alexandria University, Alexandria, Egypt

**Keywords:** Health service challenges, Post-partum family planning, Providers’ perspectives, Socio-cultural challenges

## Abstract

**Background:**

Postpartum family planning (PPFP) focuses on prevention of unintended pregnancy and closely spaced pregnancies through the first 12 months following childbirth. Adoption of family planning during the postpartum period in Egypt faces unique challenges. This study aimed to explore the socio-cultural and health service challenges related to PPFP in Alexandria, Egypt, from perspectives of family planning providers working in public settings.

**Methods:**

Three focus group discussions were conducted in the period from May to July 2017. It included 32 family planning physicians working in the family health centers and maternal and child health centers in Alexandria for 3 years or more. A discussion guide was prepared. Analysis of data was done using thematic data analysis using a deductive approach.

**Results:**

The working experience of participants ranged from 3 to 30 years. The most frequently reported reasons for unmet need for PPFP were cultural norms about the contraceptive effects of lactation and inaccurate knowledge of women about the conditions for appropriate use of the lactation amenorrhea as a contraceptive method. The most commonly cited challenge related to the quality of service was the inadequate health education services about PPFP. Lack of training and supervision of community health workers was one of the underlying causes of the perceived inadequacy of the service.

**Conclusions:**

Mass media campaigns advocating for family planning are urgently needed with full support from the government. Improvement of health education services is a must. Redistribution of family planning providers with an increase in the staff members is highly recommended.

## Introduction

Spacing births by at least 2 years can reduce maternal mortality by 30% and child mortality by 10% [1]. On the other hand, pregnancy within the first postpartum year poses the highest risk for mother and fetus leading to increased risks of preterm birth, low birth weight, small for gestational age as well as maternal and child mortalities [2, 3].

Postpartum family planning (PPFP) or postpartum contraception is defined as the initiation of contraceptive methods within the first 12 months following delivery [4]. It focuses on prevention of unintended pregnancy and closely spaced pregnancies through the first 12 month following childbirth. It can help women space their births, providing important maternal and child health (MCH) benefits [5].

Egypt has grappled with population growth for years. In 2017, the Egyptian population hit 104 million both domestically and abroad [6]. The total fertility rate, as documented in the latest Egyptian Demographic and Health Survey (EDHS 2014), increased to 3.5 children born/woman while it was only 3 in 2008 [7]. The same survey also reported that the percentage of currently married women aged 15-49 with unmet need for family planning was 12.6% [7]. A large proportion of women with unmet need for contraception is found among those in their first year after childbirth. It was reported that nearly two-thirds of these women do not want to become pregnant but are not using a contraceptive method; nearly 40 percent have the intention to use a method but have not done so [8].

In a study conducted in 2008 in rural Egypt, it was found that 25% of women receiving antenatal care conceived during breastfeeding and 29 percent of these pregnancies were unplanned [9]. Moreover, a community-based cross-sectional survey was conducted in 2016 in Alexandria, Egypt, on a sample of 1500 post-partum women revealed an estimated prevalence of unmet need for PPFP of 16.28% and it was variable across districts [10].

It was reported in many studies that the problem of unmet need for PPFP is attributed to a combination of both organizational (system related) and societal barriers. These studies depend on perspectives of providers regarding these barriers [11, 12]. Selection of the family planning providers as the target group aimed at covering all determinants of unmet need of FP as they meet challenges and organizational barriers against achieving high-quality services and at the same time they discuss with clients the societal factors that act as barriers against timely use of contraceptives.

It is a priority to help Egyptian women in achieving healthier birth intervals and increase the potential of postpartum contraceptive use and continuation. In-depth understanding of the real challenges against achieving such a goal is urgently needed.

The present study was conducted to understand the family planning providers’ perspective about organizational factors that influence their ability to provide quality PPFP care. Also, to explore sociocultural factors that contribute to non-acceptance and low contraceptive use among postpartum women. This would assist stakeholders in Egypt to achieve their targets regarding contraceptive use in general and postpartum family planning in particular.

## Methods

### Study design and target population

A qualitative research was conducted. Three focus group discussions (FGDs) (9-12 physicians each) were held during the period from May to July 2017, on a sample of family planning providers (physicians) currently working at public family planning clinics in Alexandria (family health and maternal and child health centers). They were invited by the directorate of health Affairs in Alexandria after providing a copy of the research proposal and a formal letter explaining the idea of the research and emphasizing that participation of family planning providers should be voluntary. The proposal specified that the providers’ experience in family planning in terms of the number of working years is preferred to be variable. In addition, a variability between invitees regarding the place of work was taken into consideration as Alexandria has a known variability in the social class of inhabitants of different districts which might result in different challenges for PPFP, level of coverage with health services, and quality of services. The FGDs were conducted at Alexandria Regional Center for Women’s Health and Development (ARC). They were conducted from 10 to 12 AM.

A discussion guide was prepared. It was developed based on a thorough literature review of the topic and on the results of a quantitative research in which a survey of 1500 postpartum women were interviewed to estimate the prevalence of unmet need for PPFP in Alexandria and identify its common reasons [8]. In view of the main objective of the current work to clarify challenges to PPFP both societal and organizational, a series of open-ended questions were formulated (Table 1).

**Table 1:** Discussion guide

- How do you perceive PPFP in the catchment area of your clinics? (opening question) - What are the social barriers to the use of contraceptives within 1 year postpartum? (key Q1) - What are the common organizational and service-related obstacles hindering use of contraceptives within 1 year postpartum? (key Q2) - How can we improve the rate of early postpartum contraceptive use among Egyptian women? (ending question)	

Each FGD was 90 min on average. Fifteen minutes were specified for each question and this was taken into consideration while moderating the discussion. The remaining 30 min were consumed in sharing and comparing experiences and discussing the extent to which they agree or disagree with each other.

The research team comprised of the moderator and two other researchers. The role of the moderator was to stimulate and support the flow of discussion, ask questions one after the other, redirect the track of discussion whenever indicated, but he did not function as an expert in the topic and did not present his viewpoint on the topic. The other two researchers were taking notes, observing, and documenting any non-verbal messages during the discussion. All FGDs were tape-recorded after taking permission from the participants.

Pretesting of the discussion guide was conducted in the first session as it lasted for 2 h. More time was consumed to check that all questions are clear and the exact meaning is captured by participants. Moreover, participants were encouraged to clarify questions they feel unsure about. As no dramatic modifications were done in the discussion guide, the data obtained was used as part of the results.

Before starting a group discussion, and after welcoming, an overview of the topic of PPFP and the objective of the research were explained by the researchers. The researchers confirmed to participants that their participation is voluntary, and they have the right to decline if they are not interested. In addition, they were informed that there is no right or wrong answer in this discussion. “We are interested in knowing what you perceive and get benefit from your working experience, your discussion will assist the decision makers in your field to understand the significant barriers to achieve their targets, so please feel free to be frank and to share your point of view with openness and honesty”. They were informed also that complete confidentiality and anonymity while presenting the results were ensured. This was followed by asking participants to introduce themselves. No compensation or gifts were given to the participants.

### Data analysis

Thematic data analysis with deductive approach was used. A full written transcript was prepared for the three discussions based on the record and the notes written by the two note-takers. Based on the extensiveness, frequency, and intensity of comments as observed during writing the transcript, themes were picked and given reference numbers (axial coding). The frequency of codes for all themes was calculated manually in the three groups across all individuals through grouping of key statements, ideas, and expressed views under each theme. This process was done by two persons. The most noteworthy quotes noticed in the transcript were picked to be presented to the reader to support certain themes.

To ensure reliability of analysis, cross checking of codes was performed by a third independent researcher who was given the list of codes and asked to identify sentences/groups of sentences that match each code. Code-to-sentence matching of 80% or more was necessary to ensure objective measure of views, opinions, and attitudes. The level of agreement or disagreement on different issues and opinions between participants was also taken into consideration and issues with significant disagreement were dealt with caution.

## Results

### Participants’ characteristics

A total of 32 family planning providers have participated in the FGDs with a range of 9-12 physicians in each FG. All were females as all family planning providers in the public sector in Alexandria are females; however, this is not the situation in other governorates. Their age ranged from 28 to 53 years. All were physicians with working experience ranged from 3 to 30 years and included two regional managers. Characteristics of participants are presented in Table 2.

**Table 2:** Baseline characteristics of family planning providers working at public family planning clinics in Alexandria, 2017

Baseline characteristics of participants no. (%)	
**Age in years**	
Less than 30	5 (15.6)
30 to less than 40	17 (53.1)
40 to less than 50	7 (21.9)
50-53	3 (9.4)
**Health district**	
El-Montaza	5 (15.6)
Wasat	5 (15.6)
El-Amreya	3 (9.4)
Sharq	6 (18.7)
Gharb	5 (15.6)
El-Agamy	2 (6.3)
Al-Gomrok	4 (12.5)
Borg-Elarab	2 (6.3)
**Years of experience in family planning**	
3- < 10	14 (43.8)
10- < 20	10 (31.2)
20-30	8 (25.0)

### Physicians’ perception of the rate of PPFP in their catchment areas

After agreeing upon the scientific explanation of the term “unmet need for postpartum family planning,” participants were asked about their perception about the rate of contraceptive use among women in their districts and their opinion about the findings revealed in a recent research that nearly a sixth of postpartum women in Alexandria had unmet need for FP.

Participants’ responses indicated that it is mostly true considering that this percentage might vary from place to another and from health district to the other.

*"It is not a rule as it might be higher in semi-urban and underserved areas as in El Montaza district and much lower in other districts with higher socioeconomic standards as Wassat with higher level of education and health awareness among residents,"* (a physician, El Montaza district, 15 years of experience).

Moreover, many providers and all managers expected marked reduction in the rate of unmet need soon as a result of integration of family planning services with other provided services and improvement in the quality of services.

*"This rate is about to decrease in the near future as efforts are exerted to increase coverage with high quality family planning services including the outreach services with its intensified educational components,"* (a physician, Wasat district, 23 years of experience).

### Physicians’ views about the most prevailing socio-cultural determinates for non-use of PPFP among women in their catchment areas

#### a. Misconceptions:

a.1. Dependence on lactation in the absence of menstruation (lactational amenorrhea) as a method of contraception is still considered by many women as highly effective in preventing conception for any period without criteria and there is no need to use a contraceptive till menstruation starts again. Physicians emphasized that this misperception was deeply rooted and highly prevailing because many women had self-histories of successful experience of spacing based solely on lactational amenorrhea or similar histories among their relatives and neighbors.

*"The women consider being amenorrheic with breast feeding is acting as a natural contraceptive even for one and half years after birth,"* (a physician, Gharb district, 9 years of experience).

*Some women get confident about the effect of lactation on fertility as a result of her past successful experience or that of her relative or neighbor,"* (a physician, Sharq district, 20 years of experience).

a.2. Fear from infertility and/or prolonged vaginal bleeding or spotting after using hormonal contraceptives is one of the problems frequently cited by participants.

*"Some young women have certain objections to use intrauterine devices (IUD) and at the same time they believe that hormonal methods might postpone return of fertility after discontinuation,"* (a physician, Wasat district, 6 years of experience).

*"Some women think that injectable contraceptives for more than three years might cause infertility for life,"* (a physician, Sharq district, 21 years of experience).

*"We commonly hear from clients that their husbands refuse contraceptives as they lead to prolonged bleeding and this will be inconvenient for them,"* (a physician, Sharq district, 8 years of experience).

a.3. Insertion of IUD immediately postpartum is still unaccepted by many women; however, physicians noted that this is considered a magic solution for many problems that lead to unmet need for contraception with subsequent occurrence of unintended pregnancy.

*"The high-lying uterus after delivery by caesarian section is perceived by women as a contraindication to insertion of IUD for at least three months," * (a physician, Al-Gomrok district, 8 years of experience).

*"They falsely believe that immediate insertion of IUD after delivery will fail and it will drop shortly as the uterus is full of blood,"* (a physician, Wasat district, 14 years of experience).

*"Some women and their spouses are always afraid from immediate insertion of IUD as this might cause perforation of the uterus,"* (a physician, El-Montaza district, 22 years of experience).

#### b. Phobia from the procedures of insertion of IUDs or implants:

Participants mentioned that the wide use of cesarean section as the most common mode of delivery nowadays particularly among young women is a logic explanation of declining rate of IUD use among Egyptian women.

Young women usually deliver by C.S. and they become afraid from any sort of vaginal manipulation (a physician, Gharb district, 8 years of experience).

However, IUD is one of the most effective long acting contraceptives, fear from pain at the time of its application became a common cause of refusal especially among young women (a physician, Al-Amreya district, 5 years of experience).

#### c. Cost and effectiveness of contraceptives:

High cost of contraceptives in the private sector might be a barrier to their use. The Egyptian government offers contraceptives to the citizens at a very low price. However, sometimes, there is a mistrust between the government and the population.

Some people consider contraceptives offered by the public health sector is of low effectiveness and have higher probability of side effects as compared to those from the private sector (a physician, El-Agamy district, 16 years of experience).

Three providers reported that few physicians in the private sector might spread false information regarding contraceptives in the public sector like saying that the IUD causes severe bleeding and low back pain while that applied in the private clinic is safer.

#### d. Reluctance and indifference:

There was a consensus among participants that women occasionally have some sort of reluctance to attend the clinic and indifference regarding the probability of getting pregnant irrespective of the young age of their babies who may be less than 1 year. This might exist among women who did not achieve their desired family size.

Some women attribute this negligence regarding postpartum care in general and contraceptive use in particular to their inability to attend the health facility in the morning (a physician, Al-Gomrok district, 23 years of experience).

Many postpartum women wake up late missing the timing of service after being awake all-night caring for their infants especially in the first three months (a physician, El-Montaza district, 8 years of experience).

Some participants pointed to ignorance of some women about the benefits of adequate spacing for themselves as well as their children.

Women sometimes want to achieve their reproductive role as early as possible ignoring the impact of close pregnancies upon their health and their children. Those women need education at their home (a physician, Wasat district, 12 years of experience).

#### e. Traditional methods:

Lastly, some participants highlighted that few women depend on traditional methods for contraception such as withdrawal and the safe period.

### Physicians’ perspectives about common organizational and service-related obstacles of PPFP in their catchment areas

Regarding perspectives of the participants about the health system and its role in the problem of unmet need and missed opportunity for PPFP, the discussion revealed the most significant obstacles are as follow:

#### Inadequate health education activities:

One of the major health service-related obstacles was inadequate health education services about postpartum family planning as an integral part of antenatal, natal, and postnatal care services. Motivation of women for postpartum family planning, correction of misconceptions, and providing knowledge about the best timing for use and the available suitable contraceptives are the roles of health providers as essential activities in the maternity care program. Lack of fulfillment of such activities is considered a major challenge to PPFP.

The obstetricians while following their pregnant clients during the antenatal care visits particularly in the last trimester should provide enough information and reply to all inquiries of the women regarding birth control methods (a physician, Gharb district, 7 years of experience).

During antenatal care, the obstetricians provide timely care for the mother and the fetus. They usually miss an excellent opportunity to provide education about family planning (a physician, El-Amreya district, 15 years of experience).

In many countries worldwide, before discharging women after delivery the health team usually gives an educational session for the mother through the physician, the nurse or the health educator. In Egypt, even at the highest social level this hardly ever takes place (a physician, Wasat district, 18 years of experience).

All participants who are currently working as family planning physicians ensure that educating and motivating women about using family planning early in the postpartum period is mainly the role of the community health workers either inside or outside the health facility through the outreach visits. Moreover, they should be present in the vaccination room as well as gynecology clinics to communicate with women and refer them to family planning clinics. Unsatisfactory performance in this role is considered a major obstacle to high-quality family planning services in Egypt in general.

Reluctance and inadequate supervision of community health educators are the main underlying reasons behind absence of their role in the community (a physician, Al-Gomrok district, 30 years of experience).

This also might be partially attributed to inadequate salaries and lack of incentives for such very important personnel (a physician, Sharq district, 25 years of experience).

Providers attributed this to inadequate supervision from the physicians of community health workers due to either the work overload on physicians or the sensitive relation between physicians and community health workers leading to it being unacceptable for the latter to be supervised by the physicians.

#### Missed opportunities:

Raising the issue of missed opportunity to PPFP in public settings showed that the behavior of some service providers who ask the client to go and come back when menstruating—because they are concerned about giving a contraceptive method to a pregnant woman—is considered an important cause. This practice could result in unintended pregnancies.

Till now, despite frequent training, some old providers insist on seeing menstrual blood at the time of insertion of the IUD (a physician, Borg-Elarab district, 4 years of experience).

#### Lack of resources:

Other reported barriers included low number of family planning physicians as well as the inadequate training of new ones. This results in work overload in some clinics and long waiting times. Therefore, providing the service to some but not all the clients is usually the outcome.

In some occasions, one physician is responsible for providing services in two different units. So, she would attend each clinic every other day (a physician, El-Agamy district, 3 years of experience).

Excessive workload due to shortage of family planning physicians currently working in the governmental sector along with the subsequent exhaustion of the working ones might be a cause for missing the opportunity to use a particular method especially IUDs and implants (a physician, El-Montaza district, 10 years of experience).

There was a consensus among participants that barriers related to family planning services organization and logistics do exist. Lack of sufficient sterilized equipment (organizational level) increases the amount of time clients have to wait for services, missed opportunities, or results in offering methods that do not require the use of that equipment.

The family planning outpatient clinic in the public health care setting is usually working from 8 AM to 2 PM. Moreover, the number of sterile IUD insertion sets is usually limited and runs out at 12 AM. Facing the absence of an autoclave in a few units, women were asked to go home and come back next day as there are no sterile equipment (a physician, El-Montaza district, 5 years of experience).

#### Other organizational challenges to postpartum family planning:

During the discussion, a few opinions were raised by a minority of participants and did not show much agreement from others.

A few family planning providers don’t follow the rules of effective counseling at the time of selecting the suitable and the most acceptable method for each particular woman (a physician, El-Amreya district, 12 years of experience).

A manager explained that such practice may be caused by a lack of time, work overload, or the tendency of the provider to give certain contraceptives as evaluation of their work sometimes is based on the rate of providing long-acting reversible contraceptives (LARC) rather than other methods.

IUD or implant may be inappropriately used even if not suitable for the woman in order to obtain better evaluation scores for the service (a physician, Wasat district, 23 years of experience).

Shortage of one or more method in some settings is rarely encountered (a physician, Sharq district, 7 years of experience).

Occasionally few providers insist on serving only women whose residence is within the catchment area which leads the client to leave without getting the service (a physician, Al-Gomrok district, 18 years of experience).

### Physicians’ suggested recommendations for improving PPFP use in their catchment areas

At the end of the discussions, the participants were asked to suggest recommendations that may reduce the level of unmet need for PPFP. The commonly raised recommendations by all participants are summarized as follows:

- Benefits of early postpartum use of contraceptives and correction of related misconceptions should be emphasized at different contact points especially antenatal and postnatal visits through different health education methods.
- Mass media campaigns, that were successful and effective several years ago, should be enforced again as it creates a state of public motivation.
- Dependence on traditional contraceptives especially withdrawal and safe periods is known to have a high failure rate and specific requirements. This message should be clearly explained for women and their husbands.
- Capacity building of family planning sector is a priority by providing the required facilities and equipment, and training physicians, nurses, and community health workers.
- Fair distribution of workload among physicians and nurses in different clinics, and provision of incentives on performance regardless of the method used should be ensured.
- There should be cooperation between the ministry of health, university hospitals, and the private sector for better family planning services. A suggestion of holding a family planning conference to gather the health care providers from the Ministry of Health, Faculties of medicine and private sector was raised, in order to discuss how to improve the quality of service.

## Discussion

Postpartum family planning is a priority area to be tackled by family planning programs as it was proven to benefit both national growth and population health [13]. It was a necessity to explain the barriers and challenges to PPFP in Alexandria from the perspective of the service providers which can facilitate acceptance of any interventions based on their recommendations.

In the current study, cultural norms about the contraceptive effects of lactation and inaccurate knowledge about the criteria for correct use of lactational amenorrhea as a contraceptive method were the most frequently reported reasons for the unmet need for postpartum family planning. The most commonly cited challenge related to the quality of service was the inadequate role played by health education services about PPFP which should be an integral part of antenatal, natal, and postnatal care services. Lack of training and supervision of community health workers was one of the underlying causes of such weakness. Moreover, understaffing, long waiting times, and deficient supplies were additional barriers for PPFP in public settings.

Providers were frustrated with continually being faced with clients’ misconceptions and rumors. Their spread is partially attributed to the lack of knowledge about contraceptives among many Egyptian women which is mainly obtained from low-quality sources like relatives, peers, other women attending health facilities, or irresponsible media. In addition, it was noted that private obstetricians sometimes play a role in spreading false rumors about the public family planning providers and the contraceptives used there [14].

In line with our results, a study including service providers from Egypt, Jordan, Sudan, and Yemen in 2008 revealed that high workload, inadequate training, and lack of health education activities were obstacles associated with the provision of postpartum family planning services. It also recommended a change of hospital policies to encourage counseling of partners on FP in the immediate postpartum period [15].

Incorrect use of the lactation amenorrhea method (LAM) was cited in our study as the most common challenge to PPFP. A study conducted in Kazakhstan evaluated interventions implemented to promote the lactation amenorrhea method and emphasized that programs must find more effective strategies for training providers and counseling clients regarding the three conditions required to use it appropriately and effectively, and how to use LAM as a bridge to the adoption of modern methods [16].

Other studies have shown that only a minority of clients and providers clearly understand the connection between breastfeeding, the return of menses, and fertility. In Egypt, Abdel-Tawab et al. [17] found that although 70% of pregnant women receive antenatal care, only 12% of physicians and 7% of nurses provide counseling on family planning during these visits. In Jordan, although one-third of the mothers who had a child aged 13–24 months reported that they had relied on breastfeeding as a contraceptive during the first 6 months postpartum (after an extensive media campaign promoting breastfeeding and LAM), only 7 percent knew the three essential criteria for a successful reliance on LAM [18].

The false belief of many women that they are not at risk of becoming pregnant particularly in the post-partum period was consistent with Willcox et al.’s study [19]. In Uganda, they noted that women were concerned about side effects and thought that using FP method was unnecessary during this period. These concerns should be addressed in health education programs to correct misconceptions and promote contraceptive uptake.

The majority of providers in the current study agreed that the unmet need for PPFP might exceed 20% in some districts. This figure is considered high as compared to that reported in other Egyptian studies. Sultan et al. [20], in their study which was conducted in an underprivileged district in Eastern Cairo, showed that 7.4% of studied women were having an unmet need for family planning. In another survey of married women (2016) in Nefesha Village in Ismailia, the reported prevalence of unmet need for family planning was 15.7% [21]. This percentage was 11.2% in a third study conducted at Mansoura governorate during 2015-2016 [22]. The current study investigated the problem among women during the first year postpartum rather than women during the childbearing period and this might explain providers’ expectation of a higher rate of unmet need.

The use of routine postnatal care in developing countries including Egypt in general is very low. This might be because women in the postpartum period usually neglect their own needs for postpartum visits and direct their time and efforts to their infants. There is a need to investigate the feasibility and usefulness of integration of PPFP with other services as the integrated service delivery model facilitates service provision. It may enable clients who may have come for other healthcare services to be provided with FP services/methods. Attending the health facility for an urgent reason such as infant immunization or child care in illness is an opportunity for those women [23]. This is in agreement with the World Health Organization strategies for postpartum family planning in which there is a continuum of points of contact within the health care system that can provide opportunities to integrate PPFP with maternal, newborn, and child health (MNCH) interventions during the 12-month period after childbirth. Moreover, immediate postpartum insertion of IUD after delivery is another window for those women [24].

### Limitations of the study

This qualitative study provides a valuable contribution to the body of knowledge on providers’ perspectives toward PPFP with a good representation for all Alexandria medical regions.

In Egypt, FP services are provided as a part of the basic health benefit package delivered at the primary healthcare level. Furthermore, a mix of contraceptives is part of the country’s essential drug list [25]. In addition, more than half (56.7%) of modern contraceptive users in Egypt receive the method from the public sector according to EDHS 2014 [7]; thus, providers at the public sector were the primary target population for conducting the study.

One of the limitations is that the study was confined to Alexandria which is a mainly urban governorate, and has one of the highest rates of use of modern contraception in Egypt. Consequently it may not reflect the other cultures, norms, and challenges in rural Egypt. Also, we did not have the opportunity to ask the participant providers to provide feedback on the results.

## Conclusions

Cultural beliefs regarding the contraceptive effects of lactation and the inaccurate knowledge of women about the conditions for the appropriate use of the lactational amenorrhea in preventing conception is a real challenge particularly among non-highly educated women.

Inadequate health education about PPFP is a common problem related to the quality of service. Lack of training and supervision of community health workers was one of the underlying causes of such weakness. They should receive on-site systematic training based on the updated guidelines in order to provide education to all postpartum women in both health and community settings. Moreover, mass media campaigns regarding family planning are urgently needed nowadays with full support from the government.

Building capacity for the health system in the area of family planning is a necessity. Increasing the number of skilled family planning providers together with their redistribution was recommended.

As Alexandria is an urban governorate with a relatively high level of health awareness, female education and better quality of health care, it is suspected that much higher rates of unmet need for PPFP would be found in other Egyptian governorates. Future research should target other governorates particularly rural areas to form a clearer picture of the challenges facing contraceptive uptake across Egypt.

## Data Availability

The datasets collected, used, and analyzed during the current study are available from the corresponding author on reasonable request.

## Abbreviations

C.S: Caesarian section
EDHS: Egypt Demographic and Health Survey
FGD: Focus group discussion
FP: Family planning
PPFP: Postpartum family planning
IUD: Intrauterine device
USAID: United States Agency for International Development
LARC: Long-acting reversible contraceptives
LAM: Lactation amenorrhea method
